# Beyond XX and XY, Understanding Sex Differences in Leukemia

**DOI:** 10.3390/medsci14010038

**Published:** 2026-01-11

**Authors:** Mai Mostafa, Alaa Elhaddad, Mohamed Z. Gad, Rasha Hanafi, Hanaa Rashad, Sami El Deeb

**Affiliations:** 1 Institute of Medicinal and Pharmaceutical Chemistry, Technische Universität Braunschweig, 38106 Braunschweig, Germany; 2 Pediatric Oncology Department, Children’s Cancer Hospital Egypt (CCHE-57357), Cairo 11311, Egypt; 3 Biochemistry Department, German University in Cairo, Cairo 11835, Egypt; 4 Department of Pharmaceutical Chemistry, German University in Cairo, Cairo 11835, Egypt

**Keywords:** sex differences, leukemia, risk factors, epidemiology, sex-based, epigenetics, metabolites, enigma

## Abstract

The major subtypes of leukemia show sex differences. This review summarizes current knowledge and identifies gaps regarding sex differences across acute myeloid leukemia, acute lymphoblastic leukemia, chronic myeloid leukemia, and chronic lymphoblastic leukemia in epidemiology, mortality and survival rates, risk factors, and epigenetic, metabolomic, and sex-specific patterns. Males have higher incidence and mortality rates of leukemia compared to females, emphasizing the importance of biological sex. Underreporting of sex differences in leukemia is highlighted, suggesting that sex is often overlooked as a research variable. A significant clinical observation is that women demonstrate higher overall survival rates but experience more severe treatment-related toxicity. Clinically, women tend to survive longer but experience more severe side effects. In contrast, a significant clinical observation in pediatric leukemia contradicts this enigma, suggesting that sex differences may be less pronounced during childhood. These differences play a significant role in how the disease develops. This review presents a sex-based perspective for hematological and biochemical patterns, genetic risk factors, environmental, lifestyle, and parental risk factors, epigenetics and metabolites. Furthermore, males and females might have different responses to the same toxic, environmental, and hormonal exposures. Trying to understand these disparities better based on molecular mechanisms is considered an approach for precision medicine.

## 1. Introduction

Males have higher incidence and mortality rates of leukemia than females do, highlighting the importance of biological and epidemiologic factors [1,2,3,4,5,6]. Globally, the disease burden is higher among males than females with leukemia [2]. The causes of the differences in disease burden between males versus females are still unknown, and a variety of factors, including genetic variants, epigenetics, hormones, senescence, immunity, and angiogenesis, may contribute to the explanation of this observation [7,8].

There is a significant knowledge gap regarding how sex hormones affect the progression of non-sex-specific malignancies, the increased risk of treatment-related side effects in female patients, and the effects of both donor and recipient sex on the outcome of allogeneic stem cell transplantation [9]. Remarkably, the Trialtrove database (https://citeline.informa.com/trials/results, accessed on 7 January 2026) revealed that only 472 out of 89,221 cancer clinical trials included curated sex-based comparisons, accounting for just 0.5% of all trials [10]. Underreporting may be a result of reporting subgroup analysis, where males and females are analyzed separately but not directly compared in clinical trials. Sex has received insufficient consideration in decision-making processes [11]. As of 2025, the practical management of hematological malignancies remains sex blind [11]. In light of this gap, we briefly review the current understanding and identify the knowledge gaps related to sex differences in epidemiology; mortality and survival rates; risk factors, and epigenetic, metabolomic, and sex-specific patterns.

## 2. Leukemia Epidemiology, Treatment Response, and Clinical Data

### 2.1. Sex Differences in Incidence and Mortality Rates

**Figure 1:**
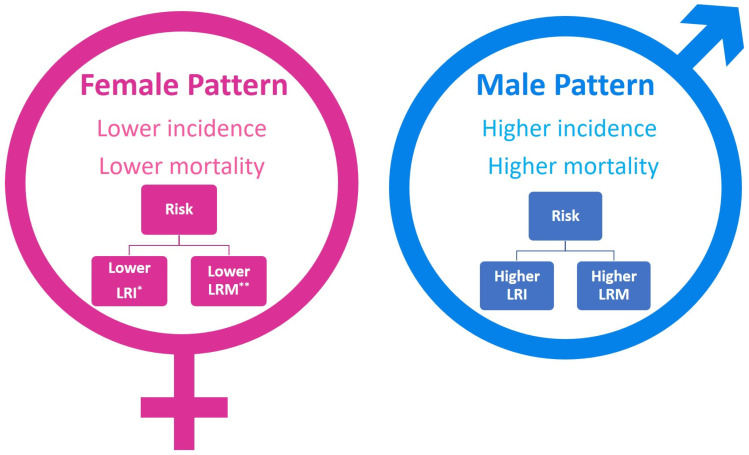
Sex specific patterns in leukemia. * LRI: global lifetime risk of incidence, ** LRM: global lifetime risk of mortality.

Globally, the incidence of leukemia is higher in male than in female patients (269,503 vs. 205,016, respectively) [12]. Leukemia mortality is higher in male patients than in female patients globally (177,818 vs. 133,776, respectively) [12]. In leukemia, the global lifetime risk of incidence (LRI) and mortality (LRM) are generally higher in males than in females [13]. The global LRI for leukemia is higher in males (0.65%) compared to females (0.54%). Similarly, males have a higher global LRM (0.44%) compared to females (0.37%) [13]. Interesting patterns arise that substantially impact disease epidemiology (Figure 1).

Leukemia has four major subtypes: ALL (acute lymphoblastic leukemia), CLL (chronic lymphoblastic leukemia), AML (acute myeloid leukemia), and CML (chronic myeloid leukemia) [14]. There are significant sex-based variations among the major leukemia subtypes. Regardless of the underlying molecular processes, males consistently have greater incidence rates and poorer survival rates in patients with AML, ALL, and CML [15]. Epidemiological characteristics such as incidence and mortality show sex differences across leukemia subtypes (Table 1).

In adults, leukemia is the 13th most diagnosed malignancy, with more than 487,000 new leukemia cases estimated in 2022. With 305,000 deaths from leukemia in 2022, it ranks as the 10th most common cause of cancer mortality globally [17,18]

Leukemia is the most common childhood cancer, accounting for 28% of cases in the United States. In 2022, leukemia (450 deaths) was the second most common cause of cancer mortality among children and adolescents under the age of 20, after brain tumors (480 deaths) in the United States, according to the American Cancer Society [16]. Globally, boys had a higher leukemia incidence rate than girls (3.31 vs. 2.20) per 100,000 population in 2021. Boys consistently had higher incidence rates than girls across all age groups: <1 year: boys, 8.05; girls, 6.13; 2–4 years: boys, 3.6; girls, 2.66; 5–9 years: boys, 2.59; girls, 1.81; 10–14 years: boys, 2.15; girls, 1.17 [19]. Boys often have greater rates than females in terms of incidence, death, and disability-adjusted life year (DALYs). Childhood leukemia is almost 1.7 times more common in males than in females, a global trend according to a large population-based study [19]. Globally, 211,080 new cases and 78,441 deaths of childhood cancers occurred in 2022. Leukemia was the most common childhood cancer, with ALL accounting for 66.73% of leukemia cases. For girls and boys, the age-standardized incidence rate of ALL was 1.69 and 2.34 per 100,000 population, respectively, based on a population registry [20].

The sex-specific age-standardized rates (ASRs) for leukemia subtypes were estimated per 100,000 for children (0–19 years) and adults (20+ years). In adults, the most common leukemia was AML worldwide (males: 38%, ASR = 3 · 1; females: 43%, ASR = 2 · 4), followed by CLL (males: 28%, ASR = 2 · 2; females: 24%, ASR = 1 · 3). The global ASR of ALL was 0.8 for males and 0.6 for females, while the ASR of CML was 1.5 for males and 1.0 for females [18]. In children, the most common leukemia was ALL worldwide (boys: 70%, ASR = 2 · 4; girls: 68%, ASR = 1 · 8), followed by AML (boys: 22%, ASR = 0 · 76; girls: 25%, ASR = 0 · 65) [18]. Leukemia is predicted to cause 720,168 incident cases and 367,804 deaths globally in 2030, based on forecasts [6]. Infant leukemia, which arises in infants aged between birth and 2 years, is a notable exception where female infants display a higher incidence [21]. In early childhood, both estrogen and androgen levels are quite low and similar for both sexes, indicating that prenatal gene expression programming may be a potential reason for the increased incidence in female infants with leukemia [22]. Caregivers and public policies need to address treatment and health care factors to lower mortality rates and minimize current disparities between sexes [7].

### 2.2. Sex Differences in Treatment Response and Overall Survival

An intriguing medical enigma in leukemia is treatment response, where women have higher overall survival rates but more severe treatment-related toxicity [15]. The observed differences in response to TKI therapy in CML patients indicate that females achieve major molecular response rates (80% vs. 45%, *p* = 0.018), despite higher treatment intolerance rates [23]. In ALL, females have better survival outcomes (42% vs. 16% of trials), despite having higher toxicity levels than males do [13]. In CLL, the incidence of treatment-induced toxicity was greater in females than in males (85% vs. 78%), especially gastrointestinal toxicity (57% vs. 42%), yet females responded better to treatment overall (83% in women vs. 71% for men), despite increased toxicity from treatment. These results suggest that better treatment outcomes may result from pharmacokinetic variations between the sexes and the action of hormones such as estrogen [24].

However, this is not always the case, as ALL pediatric patients contradict this enigma. Female pediatric patients with ALL have been reported to have a higher incidence of treatment-related toxicity, treatment delays, and deaths than males [25]. A study in ALL pediatric patients contradicts this enigma, as it revealed that female treatment toxicity and the incidence of treatment-related deaths are greater than those in males. An increase in treatment-related late effects was reported in ALL pediatric females. Females were substantially more likely than males to experience grade 3 or 4 toxicities (liver, gastrointestinal), hospital days, therapy delays, and supportive care interventions (transfusions, intravenous antibiotics) among ALL high-risk patients. The cumulative incidence of treatment-related deaths was 1.2% for males and 2.6% for females five months after treatment initiation. Male and female patients tend to have varying risk profiles, with females being more likely to develop acute and long-term treatment-related toxicities [25]. Another study in pediatric patients with T-cell ALL showed that females had shorter 5-year survival than males. Overall, female patients had more deaths than male patients. Notably, among those treated with the Capizzi methotrexate regimen, females experienced a significantly inferior 5-year disease-free survival compared to males. This difference may be explained by studies suggesting that methotrexate clearance is lower in females, resulting in higher drug levels and more toxicities [26].

Hormonal influences in childhood and adulthood may explain the enigmatic contradiction described above. Research has indicated potential protective benefits of estrogen for females [21], and sex differences may be less pronounced during childhood when estrogen and androgen levels are low [22]. Recognizing and tackling these sex differences in drug use is essential for creating more personalized and effective cancer treatments while minimizing adverse effects [27].

### 2.3. Sex Differences in Hematological and Biochemical Parameters

Hematological and biochemical profiles are crucial for determining patient prognosis before and after chemotherapy treatment [28]. Hematological parameters include complete blood count and white blood differential count, whereas biochemical parameters include renal function tests, liver function tests, serum electrolytes, and serum proteins [28]. Both hematological and biochemical patterns show sex differences across leukemia subtypes (Table 2).

Hematology parameters, such as red blood cells, hemoglobin, and platelet levels, are relatively low in pediatric B-ALL patients; however, white blood cell levels are elevated. There was a substantial difference in the biochemical profile based on sex and age between B-ALL patients, mostly males under 5 years, whereas the hematological profile did not significantly change [28].

Hematological data have shown that male adult ALL patients have more red blood cells, hemoglobin, and hematocrit, with significant mean differences compared with females [29]. Nevertheless, both sexes of ALL patients presented mean levels of red blood cells, hemoglobin, and hematocrit that were below the normal range for both sexes [30]. Notably, alterations in red blood cells are linked to the development of anemia [31]. Patients of both sexes had creatinine levels less than normal compared with standard values [29]. Patients with ALL and variations in creatinine levels have already been reported; these patients mainly exhibit kidney injury caused by hematological disease [32,33].

Compared with male patients, female patients with CML have a significantly better survival rate, which was significantly due to better survival in the low- and intermediate-risk groups. Furthermore, CML female patients had lower hemoglobin levels and higher platelet counts than did male patients. Female patients were older (51 vs. 46 years) with a smaller spleen size (3 vs. 4 cm below the costal margin) than male patients. The transplantation rate was 14% for females and 22% for males. There were no differences between males and females in WBC counts, differential, bone marrow blasts, basophils, eosinophils, and symptoms [34]. A significant difference in LDH levels was shown in patients with CML, only with respect to age [35].

In CLL, male patients had a substantially higher average WBC count, 2.92 × 10^3^/μL, than female patients did [36]. Importantly, white blood cell count, age, and sex are potential factors for patient risk assessment [37,38].

Biochemical parameters, such as the mean lactate dehydrogenase (LDH) levels in females, were found to be greater than those in males. Lactate dehydrogenase levels were significantly higher (*p* < 0.001) in all types of leukemia with age. Based on sex, Lactate dehydrogenase levels were higher in females (1722.4 ± 1557.9) compared to males (1393.5 ± 1197.6). These findings suggest that increased cellular LDH activity indicates a shift toward anaerobic metabolism and glycolysis in malignant cells, which is associated with a high turnover rate. A further practical metric for assessing the clinical and prognostic aspects of leukemia may be the measurement of LDH levels in patients [35].

**Table 2:** Hematological and biochemical sex-based patterns across leukemia subtypes.

	ALL	AML	CML	CLL
Hematological parameters	Red cells (millions/mm^3^) 3.21 males vs. 2.67 females [39]. Hemoglobin (g/dL) 9.36 males vs. 7.81 females [39]. Hematocrit (%) 28.73 males vs. 23.20 females [39].	Hemoglobin (g/dL) 9.8 males vs. 8.7 females [40]. Thrombocytes (G/L) 58 males vs. 79 females [40] Neutrophils (G/L) 1.53 males vs. 0.94 females [40] Leucocytes (G/L) 5.29 males vs. 6.86 females [40].	Hemoglobin (g/dL) 12.5 males vs. 11.7 females [34]. Platelets (10^3^/µL) 355 males vs. 459 females [34].	Hemoglobin (g/dL) 7.2 ± 1.8 males vs. 7.0 ± 1.6 females [41]. Red cells (millions/mm^3^) 2.8 ± 0.29 males vs. 2.9 ± 0.46 females [41] Platelets (10^3^/µL) 17.2 ± 11.7 males vs. 30.5 ± 21.3 females [41]
Biochemical parameters	Creatine (mg/dL) 0.54 males vs. 0.3 females [39].	LDH (x upper limit normal) levels 1.42 males vs. 1.02 females [40].	The mean LDH levels in 11–20 years (1935.9 ± 1684.2) [35] The mean LDH levels in the age group greater than 51 years (1288.9 ± 1214.3) [35]	Creatine (mg/dL) 4.23 ± 1.70 males vs. 4.44 ± 1.69 females [41]. Urea (mg/dL) 97.64 ± 28.2 males vs. 104.40 ± 28.3 females [41]. ALT (U/L) 49.1 ± 7.98 males vs. 45.6 ± 7.93 females [41].

## 3. Environmental, Lifestyle, and Parental Risk Factors

The global lifetime risk of developing (LRI) and dying from (LRM) all hematologic malignancies was 1.67% and 0.98%, respectively, in 2022. Cancer-specific analysis showed that the LRI was highest for Non-Hodgkin Lymphoma (0.73%), followed by leukemia (0.60%), while the LRM was highest for leukemia (0.41%) [13]. The causes behind the higher incidence of leukemia in males are not well understood. There has been a limited focus on how risk factors might affect males and females differently in the development of leukemia, and several epidemiological studies acknowledge sex as a risk factor but fail to detail how exposure differences might vary between the sexes [11]. The risk of developing leukemia has been linked to radiation, chemical, and pesticide exposure. The majority of research has confirmed the link between smoking and leukemia risk [42]. The primary risk factor for developing AML in males is smoking more than 30 packs of cigarettes [43]. The risk of CLL in females was found to be positively correlated with smoking. Permanent hair dye users had a slightly higher risk of leukemia development in females. The risk of CLL development increased among women employed as hairdressers and textile workers due to extensive use of dyes and bleaches [44].

The potential leukemia-related mortality risk factors, which are based on the variables provided by the GBD database (http://ghdx.healthdata.org/gbd-results-tool, accessed on 7 January 2026), are smoking, high body mass index (BMI), and exposure to carcinogens due to occupational exposure to benzene and formaldehyde [45]. The GBD database revealed that smoking had a greater impact on mortality and DALYs in males than in females. Among the 88,784.2 leukemia deaths globally associated with the risk factors listed above, smoking accounted for 72.74%, with males contributing 56.46% and females contributing 16.28%. However, leukemia-related mortality was more affected by high BMI in females [45]. Notably, among females, smoking and high BMI contribute similarly to mortality [45].

A higher risk of ALL in daughters is associated with maternal obesity during pregnancy [36]. Further research investigating this mother–daughter association may help clarify the potential etiology of sex hormone/chromosome-related ALL [46]. There was an elevated leukemia risk among the offspring of men employed in occupations in electromagnetic fields or radiation exposures, especially in males < 6 years old, but no significant relationship was detected in females [47]. The etiology of pediatric acute leukemia may be related to the domestic or garden use of pesticides three months before pregnancy [48]. In CML, males have a greater risk of developing CML, potentially because they have more target cells at risk. The hematopoietic stem cell in which BCR/ABL initiates CML is referred to as the target cell [49].

## 4. Genetic Risk Factors

Extensive research has shown varying sex-based responses to genetic stimuli. For sex-specific outcomes, two missense single-nucleotide variants (SNVs) of the *HLA-DQA1* gene, rs12722042 and rs12722039, had the largest effect sizes. The *HLA-DQA1* SNVs are linked to an increased risk of ALL in males [50]. Interestingly, the *HLA-DQA1* SNVs are in close proximity (<100 bp) to an androgen receptor binding site. Given their risk correlations with childhood ALL in boys, this finding might be significant [50]. Certain SNVs in the *ARID5B* gene are associated with ALL in both males and females, even though some SNVs have sex-specific associations. For example, the SNVs rs10740055, rs10994982, and rs6479779 are significant in females, whereas the SNVs rs10821938 and rs7923074 are significant in males [51]. Genetic variants such as deletions in the *GSTT1* gene and NQO1*2 homozygosity are associated with an increased risk of acute leukemia, with a greater impact apparent in males [52]. The persistent correlation between *SLX4IP* deletion and male sex, along with the extension of this sex bias to TAL1 locus deletion, suggests that differential illegitimate V(D)J-mediated recombination at specific loci may contribute to the consistently higher incidence rates of childhood ALL in boys than in girls [53].

*PHF6* mutations are seven times more common in males with AML than in females, whereas *PHF6* alterations exclusively occur in males with T-cell acute lymphoblastic leukemia (T-ALL) [54]. *UTX* mutations are found only in male T-ALL patients [55]. In a study of recently diagnosed AML patients, *NPM1* mutations were found in both sexes and were associated with slightly different distributions of the mutation types. Type A mutations, which result in TCTG duplication, are present in 75–80% of patients with *NPM1*-mutated AML. While 10% of all *NPM1*-mutated AML cases are caused by the second mutation, type B, which involves the insertion of CATG. Furthermore, type D, which is less common and accounts for 5% of *NPM1* gene mutations, involves the insertion of CCTG. Males were more likely to have type B mutations, whereas females were more likely to harbor types A and D mutations [56]. In adult AML patients, women are more likely than men to have a normal karyotype and *FLT3-ITD*, *DNMT3A*, *NPM1*, and *WT1* mutations, whereas complex karyotypes and *ASXL1*, *SRSF2*, *U2AF1*, *RUNX1*, or *KIT* mutations are less common [57]. The 2022 European LeukemiaNet [58] classification revealed sex differences in the percentages of patients assigned to genetic-risk groups, whereas more women were in the intermediate-risk category, and more men were in the adverse-risk group [57].

In CLL, male sex has been identified as a risk factor for both CLL development and poor survival outcomes [24,59,60]. High genetic risk and laboratory markers, including unmutated immunoglobulin heavy chain variable (IGHV) genes, mutated or deleted tumor protein 53 (*TP53*) genes, 11q deletions, elevated beta-2 microglobulin concentrations, ZAP-70 positivity, and CD38 positivity, are less common in women with CLL than in men [24].

Leukemia formation and progression may be impacted by sex-biased gene-regulatory networks and splicing events that contribute to phenotypic variations between sexes [61]. Sex influences hematopoietic stem cell (HSC) aging and leukemia development disparately in males and females. Myeloid-biased differentiation in aging males was driven by increasing myeloid cell output, while lymphoid cell output decreased in aging females. Sex-differentiated HSC aging impacts hematopoiesis, leukemogenesis, and gene functions [62].

## 5. Epigenetic Patterns in Males and Females

Dysregulation of epigenetic patterns and mutations in epigenetic modifiers that disturb normal blood cell formation play a role in the onset of various types of leukemia [63,64]. Most alterations in methylation-regulated gene expression focus on those genes that play roles in cell signaling, growth, differentiation, and programmed cell death. Methylation has proven valuable in predicting relapse possibilities and outcomes from a clinical perspective [65].

In ALL, a significant incidence of methylation is common in adolescent and young adult ALL patients, with greater methylation related to particular clinicopathologic characteristics, such as male gender and an elevated WBC count. Patients frequently exhibit abnormal methylation, particularly males, which may indicate a common pathogenic mechanism [66].

In AML, male and female DNA methylation patterns are very different, particularly in *CpG*-rich promoter regions. Almost 10% of these promoters exhibit differential methylation between sexes. Functional analysis revealed that genes with differentially methylated promoters are associated primarily with homeobox, cell development, and morphogenesis, with men having a greater number of enrichments than women do (1893 and 322 enrichments, respectively). Potential epigenetic prognostic markers were found to be sex specific, with male and female survival-significant gene sets differing by 75% [67].

Research in CLL revealed 1043 sex-related differentially methylated positions (DMPs), including 987 on the X chromosome and 56 on autosomes. These DMPs are associated with variations in gene expression between male and female CLL patients, indicating that differences in DNA methylation may contribute to sex-related differences in CLL risk. Based on published B-cell RNA-sequencing data, 18 genes covered by DMPs presented varying levels of expression in male and female CLL patients. Among these genes, *TRIB1* is an autosomal gene that has been demonstrated to suppress apoptosis and thus promote tumor growth. An epigenome-wide association analysis revealed that changes in DNA methylation may contribute to sex-related differences in the risk of developing CLL [68].

*DAPK1* gene methylation was more common in females with advanced CML than in males, indicating that the epigenetic regulation of the disease differs between the sexes. Although the exact function of DNA methylation in CML development is not fully understood, researchers could discover new treatment options for the later stages of the disease by examining the epigenetic changes associated with genes that regulate CML [69].

Epigenetic variations, such as DNA methylation and histone modification, affect gene expression differently in males and females, influencing leukemia susceptibility [65]. Co-mutational patterns differ between sexes, such as mutations in RNA splicing and epigenetic modifier genes [61]. Sex-based chromatin accessibility B cell-specific loci tend to be more likely in an open status in females and a closed status in males, suggesting that chromatin conformation contributes to sex-biased gene expression [70]. Personalized epigenetic approaches should not only incorporate genetic and molecular profiles [61], but also can help overcome challenges such as chemoresistance in cancer therapy by tailoring treatment on the basis of individual epigenetic signatures [71].

## 6. Sex-Specific Metabolites in Leukemia

“Omics” is a scientific discipline that explores the influence of genes, proteins, and metabolic pathways on disease susceptibility to enhance diagnosis, prognosis, and the development of new drugs. Advances in technology and informatics have enabled large-scale analyses of genes, proteins, and metabolites, resulting in fields such as genomics, proteomics, and metabolomics, which have improved our understanding of certain disorders [72]. In addition to the fields mentioned above, new omics approaches known as “sex omics” have been proposed to investigate sex-specific aspects of the biomedical sciences [72]. One of the most recent significant additions to -omics techniques is metabolomics, described as the qualitative and quantitative analysis of the metabolome, which is the whole set of metabolites found in cells, biological fluids, and tissues [73]. Owing to continuous advancements in the field, metabolomics seeks to offer personalized and optimal treatment plans in addition to a more rapid and precise diagnosis [74]. Taking sex differences into account could contribute to the discovery of biomarker metabolites for diagnosis, prognosis, and treatment, as well as clinical innovations aimed at improving men’s and women’s health [72].

Using newborn dried blood spots, untargeted metabolomics was performed to analyze neonatal exposure as a potential risk factor for AML. Metabolite features linked to AML were identified via sex-stratified analysis since variations in AML incidence rates indicate sex disparities. It appears that neonatal metabolomic profiles of pediatric AML risk are sex-specific because there was no overlap between the 16 predictors of AML in females and the 15 predictors in males. In males, the metabolite profile of AML predictors appears to be heterogeneous, while in females, ceramides, a class of metabolites associated with the proliferation of cancer cells, were putatively annotated as four predictors of AML. In females, breastfeeding duration was strongly associated with two metabolite predictors of AML, suggesting a potential biological link between childhood leukemia and this putative protective risk factor [75].

A comprehensive profiling of pituitary hormones and circulating sex steroids in CLL patients, both male and female, revealed a sex-specific association of these signaling molecules with treatment-free survival. The circulating hormone profiles of healthy donors and CLL patients differed significantly. Males had a lower median treatment-free survival (TFS) than females did (80 vs. 135 months, respectively). Shorter TFS in male CLL patients was associated with higher luteinizing hormone levels. An increased TFS was linked to female CLL patients with elevated levels of testosterone, dihydrotestosterone, and biologically active estrogen metabolites. CLL female patients who expressed high levels of the steroid-inactivating *UGT2B17* enzyme showed reduced TFS. These results suggest that distinct biological mechanisms are linked to the progression of leukemia in males and females and support the idea that CLL is a hormone-responsive disease [76].

It is crucial to prioritize sex considerations during the stages of research planning and implementation, ensuring that studies include both sexes as subjects and ultimately perform omics studies in both mixed-sex groups and male/female-separated cohorts [77]. However, there are clear signs of sex-based differences in the recognition of various metabolites in males and females, which could support shaping the foundation for future research [78].

## 7. Implementing Biological Insights in Clinical Settings as Personalized Medicine

Sex differences in leukemia are influenced by genetic, epigenetic, and immune responses (Table 3). Genetic factors such as loss of a sex chromosome (LOS) are common in hematological cancers. Among 868 patients with hematologic diseases, 5.1% exhibited LOS [79]. The highest frequency was observed in AML patients at 9.5%, compared to a range of 0-6% for ALL, CLL, and CML [79]. Epigenetic factors reflect the role of gender-specific *H3K27* methylation imbalance in T-cell leukemogenesis. *UTX* mutant leukemias are more sensitive to *H3K27me3* inhibitor therapy, providing new opportunities for epigenetically targeted therapy in T-ALL [55]. Males and females differ in the abundance of innate immunity cellular components [80]. Responses to cancer neoantigens are affected by normal sex variations in adaptive immunity [81,82,83,84]. Male and female disparities in cancer outcomes and treatment responses have been linked to sexual variations in innate and adaptive immune responses [21,85]. Adult females often develop stronger innate and adaptive immune responses than do males [86,87]. Sex steroids (such as estrogens, testosterone, and progesterone), which bind to hormone receptors on the surface of immune cells and modulate the function of immune system molecules, are primarily responsible for the differences in disease incidence and expression between males and females [86,88]. Steroid sex hormones influence leukemias based on sexual characteristics. Males may be more susceptible to developing ALL than females since they have lower levels of estrogen [89]. A substantial elevation in progesterone levels was observed in AML and ALL patients. Patients with AML showed a significant increase in follicle-stimulating hormone (FSH) levels relative to the control group, while ALL patients displayed a reduction in FSH levels [90]. The estradiol levels were significantly lower in patients with AML and ALL. While males have higher incidence and mortality rates in leukemia, it is crucial to highlight that leukemia is not currently categorized as hormone-regulated [90].

The potential for sex-specific risk stratification and treatment approaches, as well as the need for further research into the underlying mechanisms, are highlighted by sex differences [40]. Precision or personalized medicine (PM) concept is based on advanced molecular profiling, including genetics, to detect patient-specific alterations and tailor treatment and follow-up on an individual patient basis [91]. Hereby, each patient receives more effective treatment with fewer side effects, with the ultimate goal of prolonging survival and/or curing the condition [92]. Grasping the distinct impacts of exposure and sensitivity to hazardous stimuli in males and females would enhance the effectiveness of personalized prevention [11]. The development of PM in hematological malignancies has great potential, but it also presents significant challenges, such as data interpretation and analysis. For maximizing the benefit for our patients, it is crucial to systematically monitor and assess patient outcomes with smart trial designs as well as among real-world patients [92].

**Table 3:** Molecular mechanisms behind sex differences in leukemia.

Mechanism	Female Characteristics	Male Characteristics
Genetic Factors Sex chromosomes (XX vs. XY)	*Idic(X)(q13)* is the most frequently observed structural anomaly of the X chromosome in hematologic malignancies [93]. This chromosomal abnormality has been reported exclusively in older female patients with myelodysplastic syndromes (MDS), chronic myeloproliferative disorders, and AML [93,94].	The most common chromosomal abnormality in adult male blood cells is mosaic loss of chromosome Y (mLOY) [95]. Leukocyte mLOY is a risk factor for hematological malignancies, such as myelodysplastic syndrome, ALL, and AML [96]. mLOY functionally drives leukemogenesis and clonal hematopoiesis [97].
Epigenetic factors	The epigenetic modifier lysine demethylases *KDM6A* (*UTX*), which escapes X-chromosome inactivation, is expressed at higher levels in females [98]. In T-ALL *KDM6A* has been identified as a tumor suppressor [55].	Notably, *UTX* mutations were observed exclusively in male T-ALL patients [55]. *UTX* is the first identified X-linked tumor suppressor gene that may partially account for the skewed gender distribution (3:1) toward males in T-ALL on a genetic level [55].
Innate immunity	Females have higher numbers of macrophages and neutrophils, resulting in greater phagocytic activity [80].	Males have higher numbers of natural killer cells than females [80].
Adaptive immunity	Females have higher levels of circulating B cells [81,82,83,84]. Higher absolute CD4 cell count and a higher fraction of activated CD4^+^ T cells [81,82,83,84].	Males have higher levels of CD8 T cells [81,82,83,84]. Lower CD4:CD8 ratio than females [81,82,83,84].
Sex hormones	Females have higher levels of progesterone and estrogen [99]. Estrogen receptors are detected in immune cell types such as lymphocytes, monocytes, and macrophages [99], which have protective properties and immunostimulant effects [100]. Progesterone could be effective in growth-inhibiting *NALM6* cells by decreasing cell viability and lowering ROS levels [101].	Males have higher testosterone concentrations [99]. Testosterone has immunosuppressive effects [102]

## 8. Conclusions

Despite the obvious sex-based patterns in leukemia, there are still significant knowledge gaps. Additional research is needed to clarify the mechanisms underlying sex-based disparities in disease biology. The inclusion of sex-specific factors could improve the prediction of outcomes and treatment approaches in leukemia patients. We encourage researchers not only to perform sex-stratified analyses in their ongoing studies but also to design sex-based clinical trials. Considering sex differences will pave the way for personalized medicine.

## Figures and Tables

**Table 1:** Sex based differences in epidemiology across leukemia subtypes.

	ALL	AML	CML	CLL
Incidence Estimated new cases by sex, United States, 2025 [16]	3450 males vs. 2650 females.	12,060 males vs. 9950 females	5610 males vs. 3950 females.	14,340 males vs. 9350 females.
Mortality Estimated deaths by sex, United States, 2025 [16]	720 males vs. 680 females.	6130 males vs. 4960 females.	740 males vs. 550 females.	2810 males vs. 1650 females.

## Data Availability

No new data were created.
